# Surgeon experience in glioblastoma surgery of the elderly—a multicenter, retrospective cohort study

**DOI:** 10.1007/s11060-023-04252-3

**Published:** 2023-01-31

**Authors:** Johannes P. Pöppe, Lukas Machegger, Jürgen Steinbacher, Harald Stefanits, Sophie Eisschiel, Andreas Gruber, Matthias Demetz, Barbara Ladisich, Theo F.J. Kraus, Serge Weis, Sabine Spiegl-Kreinecker, Alexander Romagna, Christoph J. Griessenauer, Behnam Rezai Jahromi, Ilari Rautalin, Mika Niemelä, Miikka Korja, Christoph Schwartz

**Affiliations:** 1 Department of Neurosurgery, University Hospital Salzburg, Paracelsus Medical University, Salzburg, Austria; 2 Department of Neuroradiology, University Hospital Salzburg, Paracelsus Medical University, Salzburg, Austria; 3 Department of Neurosurgery, Neuromed Campus, Kepler University Hospital, Johannes Kepler University, Linz, Austria; 4 Institute of Pathology, University Hospital Salzburg, Paracelsus Medical University, Salzburg, Austria; 5 Division of Neuropathology, Department of Pathology and Molecular Pathology Kepler University Hospital, Johannes Kepler University, Neuromed Campus, Linz, Austria; 6 Department of Neurosurgery, München Klinik Bogenhausen, Munich, Germany; 7 Department of Neurosurgery, Helsinki University Hospital, University of Helsinki, Helsinki, Finland

**Keywords:** Elderly, Extent of resection, Glioblastoma, Morbidity, Outcome, Surgeon experience

## Abstract

**Purpose:**

To assess the impact of individual surgeon experience on overall survival (OS), extent of resection (EOR) and surgery-related morbidity in elderly patients with glioblastoma (GBM), we performed a retrospective case-by-case analysis.

**Methods:**

GBM patients aged ≥ 65 years who underwent tumor resection at two academic centers were analyzed. The experience of each neurosurgeon was quantified in three ways: (1) total number of previously performed glioma surgeries (lifetime experience); (2) number of surgeries performed in the previous five years (medium-term experience) and (3) in the last two years (short-term experience). Surgeon experience data was correlated with survival (OS) and surrogate parameters for surgical quality (EOR, morbidity).

**Results:**

198 GBM patients (median age 73.0 years, median preoperative KPS 80, IDH-wildtype status 96.5%) were included. Median OS was 10.0 months (95% CI 8.0–12.0); median EOR was 89.4%. Surgery-related morbidity affected 19.7% patients. No correlations of lifetime surgeon experience with OS (P = .693), EOR (P = .693), and surgery-related morbidity (P = .435) were identified. Adjuvant therapy was associated with improved OS (P < .001); patients with surgery-related morbidity were less likely to receive adjuvant treatment (P = .002). In multivariable testing, adjuvant therapy (P < .001; HR = 0.064, 95%CI 0.028–0.144) remained the only significant predictor for improved OS.

**Conclusion:**

Less experienced neurosurgeons achieve similar surgical results and outcome in elderly GBM patients within the setting of academic teaching hospitals. Adjuvant treatment and avoidance of surgery-related morbidity are crucial for generating a treatment benefit for this cohort.

**Supplementary Information:**

The online version contains supplementary material available at 10.1007/s11060-023-04252-3.

## Introduction

Glioblastoma (GBM) CNS World Health Organization (WHO) grade 4 is the most common, malignant intrinsic brain tumor in adult patients [1, 2] with a reported median age at initial diagnosis of approximately 65 years [2]. Elderly high-grade glioma patients show a worse overall survival (OS) compared to younger patients, with reduced ability to tolerate therapeutic interventions and higher rates of unfavorable biomarker status [3, 4]. Only moderate effects of gross total resection (GTR) – especially compared to biopsy alone – on OS have been demonstrated in patients older than 65 years [5–10]. Considering this background any surgery-related morbidity in elderly GBM patients might mitigate the potential benefits of aggressive surgical treatment [8, 10–15].

Prior studies have shown that a center’s higher caseload/surgeon volume for surgically treated oncological patients leads to improved outcome [16, 17], an association that has been substantiated for several neurosurgical procedures [18–22]. Two different studies have previously reported a favorable outcome for GBM patients if surgery is performed by specialist neurooncology neurosurgeons [23] and if patients are treated at academic/high-volume centers [23, 24]. Here we evaluated the impact of surgeon experience on elderly (i.e. age ≥ 65 years at diagnosis) GBM patients’ outcome after microsurgical tumor resection.

We hypothesized that greater individual surgeon’s experience leads to (1) improved OS, (2) improved EOR, and (3) decreased surgery-related morbidity/mortality.

## Methods

### Patient selection and histopathology

We identified GBM patients with an age ≥ 65 years at the date of initial surgery, who had undergone microsurgical resection as primary treatment at two university hospitals between 02/2012 and 12/2016 (Center A) and 09/2006 to 06/2021 (Center B). Exclusion criteria were defined as emergency surgery, biopsy only, palliative care only as well as patients with known secondary GBMs. The two hospitals have a combined catchment area of approximately 2,250,000 inhabitants. Annual caseloads of glioma surgeries were approximated to be ~ 120 (Center A) and ~ 50 (Center B).

Histopathological grading was performed according to the WHO classification of tumors of the central nervous system valid at the time of diagnosis [25, 26]. Clinical data extraction was performed from electronic medical records and included patient characteristics such as age, sex, preoperative Karnofsky Performance Scale (KPS), tumor location and leading symptoms.

All patients/legal guardians gave written informed consent prior to all surgical procedures and adjuvant treatments. The State Review Board approval was obtained for the retrospective GBM databases this study was conducted upon (IRB-Number 415-E/2247/2-2017).

### Course of treatment, follow-up, morbidity and mortality

Fluorescence-guided surgeries with 5-aminolevulinic acid (5-ALA) as well as intraoperative ultrasound and neuronavigation were routinely used at both centers; one center had additional access to intraoperative MRI for selected cases (Center A).

Postoperatively, patients were evaluated at the neurosurgical outpatient clinic, usually within two to four weeks, and underwent magnetic resonance imaging (MRI) at 3-months intervals over the course of follow-up. All adjuvant treatment and surgery-associated morbidity/mortality were recorded; surgery-related morbidity and mortality were defined as death within 30 days after surgery, new or worsening preoperative neurologic deficits/epilepsy, symptomatic intracranial hemorrhage, pulmonary embolism, organic psychosyndrome (OPS), cerebrospinal fluid fistulas requiring revision surgery and local/systemic infections. OS was calculated until last clinical follow-up or death; the date of last follow-up was November 30th, 2021.

### Surgeon experience data

We determined all corresponding surgical teams for the recorded tumor resections. Each surgical team consisted of two neurosurgeons, at least one of whom held a neurosurgical board certification. For each lead surgeon, we calculated the total number of previously performed cranial microsurgical glioma resections (lifetime experience), the number of surgeries performed in the last five years (medium term) and two years (short term) for each index surgery. If we were unable to obtain this information, these cases were excluded.

### Imaging analysis

For the majority of cases, preoperative and early postoperative (≤ 72 h after surgery) magnetic resonance imaging (MRI) data were available. All available pre- and immediate postoperative MRIs were centrally re-evaluated to determine the EOR by members of the Institute of Neuroradiology (LM, JS) blinded to the patients’ clinical outcome and surgical teams (Supplement 1). The difficulty level of all performed surgeries was assessed by the Milan Complexity Scale (MCS), i.e. with regard to major brain vessel manipulation, posterior fossa location, cranial nerve manipulation, eloquence and tumor size [27].

### Statistical analysis

Survival analyses were performed using the Kaplan-Meier method. Surgeon experience for all three time spans (i.e. lifetime, medium- and short-term experiences) was correlated with OS, EOR and surgery-related morbidity and mortality (Supplement 1). Given that it has been reported that GBM outcome improved during the time frame of this study [3], we performed separate outcome analyses for two dichotomized periods (i.e. period 1 (≤ 2013) and period 2 (2014 and onwards) in an effort to rule out the surgery date itself as a potential confounding factor.

## Results

198 GBM patients (112 males) met inclusion criteria. Median age at tumor resection was 73.0 years (range 65–88), median preoperative KPS was 80 (range 40–100). Most common symptoms leading to diagnosis were epileptic seizures (n = 35/198), motor deficits (n = 30/198) and headache (n = 25/198). The most common tumor locations were the temporal (n = 65) and frontal lobe (n = 55). Histopathological analyses revealed an IDH1/2 wildtype status in 191/195 (97.9%) tumors (Table 1).

**Table 1:** Patient and tumor characteristics

Factor	All patients	Center A (n = 64)	Center B (n = 134)
Sex (males)	110/198 (55.6%)	39/64 (60.9%)	71/134 (53.0%)
Median (range) age at surgery in years	73.0 (63.0–88.0)	73.5 (64.0–86.0)	72.0 (63.0–88.0)
Mean ± SD age at surgery in years	72.6 ± 5.1	73.0 ± 5.1	72.5 ± 5.1
Median (range) preoperative KPS in years	80 (40–100)	80 (40–100)	80 (50–100)
Mean ± SD preoperative KPS	79.9 ± 13.3	80.0 ± 11.7	79.6 ± 16.8
Right hemisphere	111/198 (56.1%)	37/64 (57.8%)	74/134 (55.2%)
Frontal lobe	55/198 (27.8%)	16/64 (25.0%)	39/134 (29.1%)
Temporal lobe	65/198 (32.8%)	19/64 (29.7%)	46/134 (34.3%)
Parietal lobe	24/198 (12.1%)	11/64 (17.2%)	13/134 (9.7%)
Occipital lobe	10/198 (5.1%)	5/64 (7.8%)	5/134 (3.7%)
Frontotemporal lobes	7/198 (3.5%)	4/64 (6.3%)	3/134 (2.2%)
Temporooccipital lobes	7/198 (3.5%)	3/64 (4.7%)	4/134 (3%)
Parietooccipital lobes	12/198 (6.1%)	3/64 (4.7%)	9/134 (6.7%)
Other	18/198 (9.1%)	3/64 (4.7%)	15/134 (11.2%)
Median (range) tumor volume in cm^3^	29.6 (0.2-167.4)	28.6 (0.2–92.1)	31.5 (0.5-167.4)
Mean ± SD tumor volume in cm^3^	35.7 ± 27.5	29.8 ± 22.4	38.5 ± 29.3
IDH 1/2 wildtype*	191/195 (97.9%)	62/63 (98.4%)	129/132 (97.7%)
MGMT-promoter methylated*	96/156 (61.5%)	49/64 (76.6%)	47/92 (51.1%)
TERT promoter mutated*:	69/122 (56.6%)	10/46 (21.7%)	59/76 (77.6%)

*IDH* isocitrate dehydrogenase, *KPS* Karnofsky Performance Status, *MGMT* methylguanine-DNA methyltransferase, *SD* standard deviation, *IQR* Interquartile range, *TERT* telomerase reverse transcriptase

*Data not available for all tumors

### Treatment, clinical outcome and extent of resection

**Fig. 1:**
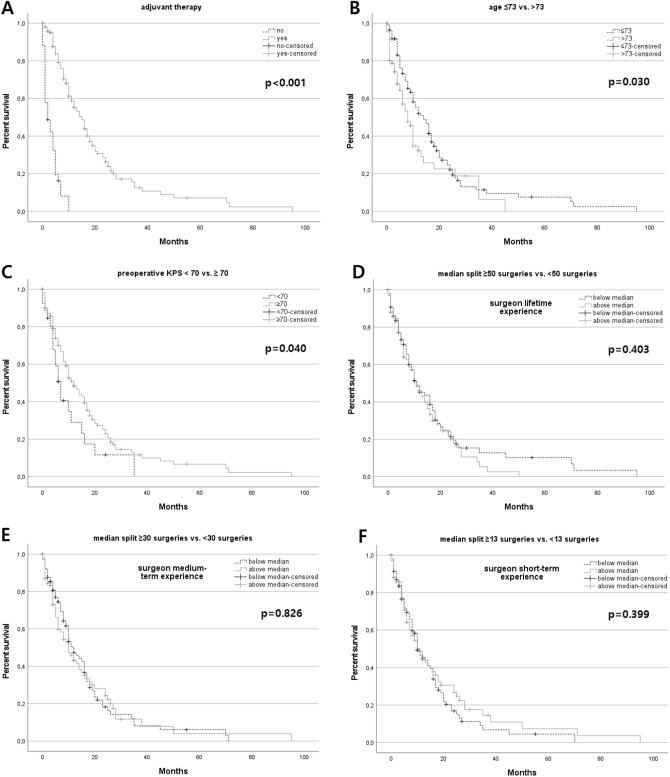
Kaplan–Meier survival analyses

Median OS was 10.0 months (95%CI 8.0–12.0), the median EOR of 89.4% (range 12.7–100). Gross total resection (EOR ≥ 95%) was recorded for 57/171 (33.3%) patients. Most patients 154/198 (77.8%) received adjuvant treatment most frequently consisting of concomitant radiochemotherapy (RTx/CTx) followed by adjuvant temozolomide [28] (Table 2). Adjuvant therapy correlated with improved median OS of 15.0 (95%CI 11.8–18.2) vs. 2.0 (95%CI 0.0–4.2) months (p < .001) (Fig. 1A).

**Table 2:** Treatment, clinical outcome and extent of resection

Extent of resection*	
Median (IQR) EOR%	89.4 (78.3–96.5)
EOR ≥ 95%	57/171 (33.3%)
EOR 70–94%	83/171 (48.5%)
EOR < 70%	31/171 (18.1%)
Adjuvant treatment	
Adjuvant treatment	154/198 (77.8%)
RTx only	22/198 (11.1%)
CTx only	7/198 (3.5%)
RTx/CTx	125/198 (63.1%)
Best supportive care only	44/198 (22.2%)
Clinical outcome	
Median (95%CI) PFS in months	8.0 (6.1–9.9)
Median (95%CI) OS in months	10.0 (8.0–12.0)
Age and KPS adjusted clinical outcome	
Median OS for preoperative KPS ≥ 70 in months (95%CI)	12.0 (9.2–14.8)
Median OS for preoperative KPS < 70 in months (95%CI)	7.0 (4.8–9.2)
Median OS for age ≤ 73 years in months (95%CI)	14.0 (10.4–17.6)
Median OS for age > 73 years in months (95%CI)	8.0 (5.7–10.3)
Surgery-related data	
Median (IQR) MCS score:	3.0 (1.0–4.0)
Mean ± SD MCS score:	2.6 ± 1.6
Median duration of surgery (skin-to-skin time) in minutes (range)	194.5 (60.0-470.0)

*CTx* chemotherapy, *EOR* extent of resection, *IQR* Interquartile range, *MCS* Milan Complexity Scale, *OS *overall survival, *PFS* progression-free survival, *RTx* radiotherapy, *SD* standard deviation,

*Immediate postoperative MRI not available for all patients

Surgery-related morbidity and mortality was seen in 39/198 (19.7%) including 8/198 (4.0%) deaths. The most frequent surgery-related morbidity consisted of either new neurological deficits (n = 37), most commonly a new hemiparesis (n = 12) or worsening of a preexisting hemiparesis (n = 7), symptomatic postoperative hemorrhage (n = 13), and OPS (n = 12). Patients with surgery-related morbidity had worse OS (median 10.0 (95%CI 7.0–13.0) vs. 11.0 (95%CI 7.9–14.1) months; mean 10.4 ± 1.2 vs. 17.9 ± 2.0 months) not statistically significant (P = .051). Patients with surgery-related morbidity were less likely to receive adjuvant treatment (P = .002); 35.9% of patients with surgery-related morbidity did not receive adjuvant treatment vs. 12.9% of cases without surgery-related morbidity. Thus, surgery-related morbidity was a significant predictor for withholding further treatment (P = .019). Accordingly, a higher postoperative disability as assessed by modified Rankin Scale correlated with worse OS (p ≤ .001). Higher preoperative KPS (≥ 70 vs. < 70; P = .040) and younger age (≤ 73 years; P = .030) were associated with improved OS (Fig. 1B C). Tumor volume (P = .146) and EOR (P = .469) did not correlate with OS. MGMT-promoter methylation did not correlate with improved OS (P = .632) in our patient cohort; furthermore, MGMT-promoter methylation did not associate with surgery-related morbidity (P = .599) and performance of adjuvant treatment (P = .399).

Additional analyses showed that there was a significant difference with regard to median EOR between centers (86.8% (IQR 62.7–95.2) vs. 90.5% (IQR 81.5–97.2); p = .041) but also with regard to the estimated median MCS (4.0 (IQR 2.0–4.0) vs. 2.0 (IQR 1.0–4.0); P < .001) (Table 2). No significant differences with regard to surgery-related morbidity frequencies (P = .846), PFS (P = .669), and OS (P = .143) between the two centers were noted in univariate analyses (Table 1). Differences in OS between periods 1 and 2 (9.0 (95%CI 7.2–10.8) vs.13.0 (95%CI 9.4–16.6) months were not statistically significant (P = .231). Also, adjuvant treatment frequencies between inclusion periods 1 (77.9%) and period 2 (77.7%) (P = .220) remained similar. 

### Surgeon experience data

We identified 37 different neurosurgeons who had performed the analyzed glioma surgeries in this series; 18 (48.7%) at Center A and 19 (51.4%) at Center B (Table 3), in 172/198 (86.9%) cases the lead neurosurgeon held a board certification at the time of tumor resection. On average 61.5 ± 54.6 (median 50, range 0–232) glioma surgeries had been performed before the index surgery by the lead surgeon. The respective average numbers for medium-term and short-term experience were 30.9 ± 22.8 (median 30, range 0–96) and 13.8 ± 9.8 (median 13, range 0–41).

**Table 3:** – Impact of surgeon experience on outcome parameters (dichotomized analyses)

Lifetime experience and overall survival		
Experience data	Overall survival	
≥ 50 surgeries	11.0 (95%CI 7.9–14.1) months	*P = .909*
< 50 surgeries	11.0 (95%CI 6.8–15.2) months	
Lifetime experience and extent of resection		
Experience data	Median (range) extent of resection	
≥ 50 surgeries	90.3% (IQR 82.3–90.3)	*P = .545*
< 50 surgeries	89.3% (IQR 77.1–96.1)	
Lifetime experience and surgery-related morbidity		
Experience data	Cohort percentage of surgery-related morbidity	
≥ 50 surgeries	24/96 (25.0%)	*P = .132*
< 50 surgeries	15/93 (16.1%)	
Medium-term experience and overall survival		
Experience data	Overall survival	
≥ 30 surgeries	10.0 (95%CI 6.3–13.7) months	*P = .111*
< 30 surgeries	12.0 (95%CI 8.8–15.2) months	
Medium-term experience and extent of resection		
Experience data	Median (range) extent of resection per cohort	
≥ 30 surgeries	90.6% (IQR 81.0–97.0)	*P = .415*
< 30 surgeries	88.8% (IQR 75.3–96.5)	
Medium-term experience and surgery-related morbidity		
Experience data	Cohort percentage of and surgery-related morbidity	
≥ 30 surgeries	25/99 (25.3%)	*P = .234*
< 30 surgeries	17/93 (18.3%)	
Short-term experience and overall survival		
Experience data	Overall survival	
≥ 13 surgeries	11.0 (95%CI 7.5–14.5) months	*P = .241*
< 13 surgeries	10.0 (95%CI 7.0Gross-total resection outcomes in an elderly population with glioblastoma: a SEER-based analysis. Clinical article12.9) months	
Short-term experience and extent of resection		
Experience data	Median (range) extent of resection per cohort	
≥ 13 surgeries	89.2% (IQR 77.4–95.7)	*P = .356*
< 13 surgeries	91.0% (IQR 80.6–98.0)	
Short-term experience and surgery-related morbidity		
Experience data	Cohort percentage of surgery-related morbidity	
≥ 13 surgeries	23/101 (22.8%)	*P = .771*
< 13 surgeries	20/95 (21.1%)	

*IQR* Interquartile range

### Impact of surgeon experience on outcome parameters

Lifetime surgical experience as a continuous variable did not show a significant correlation with OS (P = .693), EOR (P = .693), or occurrence of surgery-related morbidity (P = .435). These results were corroborated by the respective correlation analyses for medium-term (OS (P = .386), EOR (P = .542) and surgery-related morbidity (P = .530)) as well as short-term (OS (P = .499), EOR (P = .555) and surgery-related morbidity (P = .450)) experiences.

Similarly, correlation analyses with the dichotomised data did not demonstrate any significant correlation of lifetime experience (OS (P = .909), EOR (P = .545) and surgery-related morbidity (P = .132)), medium-term experience (OS (P = .111), EOR (P = .415) and surgery-related morbidity (P = .234)) and short-term experience (OS (P = .241), EOR (P = .356), surgery-related morbidity (P = .771)) (Fig. 1D, E and F and Table 3).

In a multivariable cox regression model, adjuvant therapy remained the only significant predictor for improved OS (P < .001; HR = 0.064, 95%CI 0.028–0.144), neither lifetime surgeon experience nor medium-term and short-term experiences correlated significantly with patient survival (Table 4).

**Table 4:** Cox proportional hazard model for overall survival

Factor	P-value	Hazard ratio	95% Confidence interval	
			Lower bound	Upper bound
Age	0.694	0.990	0.944	1.039
EOR (continuous variable)	0.288	0.991	0.975	1.007
EOR extent of resection (< 70%, 70–94%, ≤ 95%)	0.423	1.225	0.746	2.011
Lifetime surgeon experience (continuous variable)	0.197	0.991	0.978	1.005
Medium-term surgeon experience (continuous variable)	0.914	0.998	0.954	1.044
Short-term surgeon experience (continuous variable)	0.455	1.033	0.949	1.124
Lifetime surgeon experience (median split)	0.430	1.372	0.625	3.011
Medium-term surgeon experience (median split)	0.449	0.710	0.293	1.722
Short-term surgeon experience (median split)	0.701	1.183	0.502	2.784
Milan complexity scale	0.176	1.108	0.955	1.285
Adjuvant therapy	**< 0.001**	0.064	0.028	0.144
Preoperative KPS (continuous variable)	0.519	0.991	0.964	1.019
Preoperative KPS (< 70 vs. ≥70)	0.930	1.051	0.348	3.172
Surgery-related morbidity	0.817	1.081	0.557	2.101

*EOR* Extent of resection, *KPS* Karnofsky performance scale

Further in-depth analyses showed no correlation between MCS and surgery-related morbidity (P = .465) or the surgeons’ experience (P = .132); thus more experienced surgeons did not operate tumors of an estimated higher difficulty. Furthermore, the prognostic factors (i.e. patient age, KPS, adjuvant treatment) for improved OS were equally distributed within surgeon experience median split subgroups (Supplement 2).

## Discussion

The key findings of our study were that (1) less experienced neurosurgeons achieve similar surgical results with regard to clinical and radiological outcome in elderly GBM, IDH-wildtype patients, and (2) avoidance of surgery-related morbidity and performance of adjuvant treatment after tumor resection are crucial for generating a treatment benefit.

Compared to other studies, which often use the total provider volume and/or the annual surgeon volume as a surrogate parameter to evaluate volume-outcome associations in neurosurgery [20, 21], [29–31], we conducted a case-by-case analysis for each individual surgeon’s experience at the time of surgery. Our main hypothesis, that greater surgeon experience provides better clinical and surgical outcome, was falsified. This was consistent concerning OS, EOR and surgery-related morbidity. Contrary to our findings, in a study of younger patients (median age 54 to 57 years) with high-grade gliomas WHO grade 3 and 4, an improved OS and a lower 30-day mortality has been shown for specialist surgical neurooncologists compared to general neurosurgeons [23]. Thus, a positive effect of a surgeon’s experience in a younger cohort with supposedly more IDH-mutant gliomas seems to be evident [23]. The minor influence of surgeon volume on patient outcome in our cohort, most likely reflects the fatal course of GBM, IDH-wildtype in patients of higher age. This leads to the assumption that in elderly GBM, IDH-wildtype patients the efficacy of surgery itself is possibly limited to facilitate adjuvant therapy. This aspect underscores that a surgeon´s individual experience only plays a minor role for outcome in elderly GBM patients, because surgery itself – no matter how extensive/well-performed – has no significant influence on clinical outcome. Of note, MGMT-promoter-methylation status did not correlate with OS and performance of adjuvant treatment in our data. This somewhat contradictive aspect regarding MGMT status and survival in elderly GBM patients has also been recorded before [8].

On the other hand, no objectifiable difference in surgical quality between experienced and less experienced surgeons could be documented since we found no difference in EOR, surgery-related morbidity and mortality analyses with regard to surgeon experience. Other studies on surgeon experience in brain tumor patients have shown a reduction in 30-day mortality after brain tumor resection by more experienced surgeons [31], and improved early postoperative outcome measures for annual and five-year interval caseloads [18] whereas similar results could be achieved by less experienced surgeons compared to high-volume surgeons in very old meningioma patients [19]. In our cohort, any surgery which was performed by a less experienced surgeon was supervised by an assisting attending. This academic educational system seems to offer stable surgical quality as measured in EOR and complication rates, even in absence of an experienced lead surgeon. Although we do not believe that GBM surgery in elderly patients should be left to inexperienced residents, we did find reasonable evidence, that under experienced guidance less experienced surgeons can perform these surgeries without compromising clinical outcome.

Even though patients older than 65 years represent about half of all GBM patients [2], patients older than 70 years were excluded from some of the most important therapy defining studies [28], leaving the treatment of elderly GBM patients to case-by-case decisions. Thus, a decent approach to define appropriate treatment algorithms is necessary and first guidelines on the topic have been published [32, 33], focusing on the efficacy of cytoreduction apart from GTR only paradigms. Although a benefit of GTR in GBM patients has been confirmed by many studies [34–37], data on the impact of EOR on outcome in elderly (≥ 65), often fragile patients are scarce and less specific, but favor resection over biopsy only protocols [4, 8, 10, 12, 38, 39]. A survival benefit for any microsurgical glioma surgery compared to biopsy only in IDH-wildtype GBM patients – regardless of the extent of resection – has been reported [39, 40] as well as a survival benefit for GTR compared to partial/subtotal resection and biopsy only in a recent study with a large cohort of GBM patients older than 65 years [8]. Since GBM patients who only underwent biopsy were excluded from our study, we cannot draw a reasonable conclusion in favor of glioma resection in elderly GBM patients. However, according to our data, the extent of resection itself seems less critical in this specific patient subgroup. Thus, in elderly GBM patients the avoidance of complications to enable adjuvant treatment is probably more important than maximizing EOR at any cost. This point of view is reflected by several other authors [11, 15, 33, 41] and substantiated here for elderly, supposedly more fragile GBM, IDH-wildtype patients, since the overwhelming influence of maximized EOR has been shown to be of prognostic relevance in multiple prior studies on GBM in more age-balanced cohorts [34–36]. Moreover, it can be stated that the avoidance of complications adds to the quality of life in the last months of an elderly GBM patient – which is obviously paramount.

In summary, the two highly significant findings of this study with regard to clinical outcome are, firstly, the importance of adjuvant therapy for survival and, secondly, avoidance of surgery-related morbidity. This aspect is in line with prior data, in which a survival benefit for GBM patients treated at academic and high-volume centers compared to low volume/non-academic facilities could be shown [24]. Thus, the primary importance is not the experience level of the surgeons, but the impact of adjuvant treatment, which might be more utilized in academic and high-volume facilities.

## Limitations

In order to most reliably and objectively assess the effect of surgeon experience on the chosen outcome parameters, we opted for a case-by-case analysis approach. Nonetheless, it remains an immanent limitation that no two surgeries are alike. In the best effort to rule out potential bias factors, we retrospectively scored the assumed complexity of the surgery by the MCS and checked for any imbalances of known prognostic factors for improved OS within the analyzed subgroups. In addition, due to the limited case number there is potential risk for type 2 errors.

Furthermore, all surgeries were supervised by at least one board-certified neurosurgeon and the degree of help received by a younger colleague during the tumor resection could not be retrospectively verified by the available surgical reports. Board certification of the lead surgeon per se did not correlate with patient outcome (data not shown).

## Conclusion

According to our data, surgical resection of GBM, IDH-wildtype in patients aged ≥ 65 years may be safely performed by less experienced surgeons in the setting of an academic teaching hospital – under supervision of more experienced neurosurgeons – without compromising clinical outcome parameters. In this often fragile patient cohort, avoidance of postoperative morbidity and performance of adjuvant treatment remain most significant adjustable predictors for improved clinical outcome and should be the main focus of any surgical intervention. The observed limited influence of surgeon volume on patient outcome most likely reflects the fatal course of GBM, IDH-wildtype in patients of higher age.

## Supplementary Information

Below is the link to the electronic supplementary material.

Supplementary material 1 (DOCX 13.7 kb)

Supplementary material 2 (DOCX 12.6 kb)

## Abbreviations

CSF: Cerebrospinal fluid
CTx: Chemotherapy
EOR: Extent of resection
GBM: Glioblastoma
IDH: Isocitrate dehydrogenase
KPS: Karnofsky performance scale
MCS: Milan complexity scale
MGMT: O(6)-methylguanine-DNA methyltransferase
MRI: Magnetic resonance imaging
OPS: Organic psychosyndrome
OS: Overall survival
PFS: Progression-free survival
RANO: Response assessment in neuro-oncology
RTx: Radiotherapy
TERT: Telomerase reverse transcriptase
TMZ: Temozolomide
WHO: World health organisation
