# Supplementary material for: Surgeon experience in glioblastoma surgery of the elderly—a multicenter, retrospective cohort study
Source: J Neurooncol. 2023 Jan 31;161(3):563–72. doi: 10.1007/s11060-023-04252-3 (PMC9992256; doi:10.1007/s11060-023-04252-3)
Supplement: Supplementary file 1 — Supplementary material 1 (DOCX 13.7 kb) [file 11060_2023_4252_MOESM1_ESM.docx]

**Supplement 1: Imaging and statistical analysis**

**Imaging Analysis**

The "grow from seeds" algorithm of the 3D Slicer software (version 4.11) was used for segmentation. A volume in the lesion was randomly marked, as well as the approximate borders of the lesion on three slices in the three spatial directions. From this, the software calculated the volume of the lesion. Rare artifacts of the algorithm have been corrected by hand.

**Statistical analyses**

Statistical analyses were performed with SPSS 27 (IBM Statistics); the significance level was set as p≤0.05 (95% CI).

**Survival data**

Adjuvant therapy, preoperative KPS ((≥70 vs <70), age (median split groups, ≤73 years) and surgery-related morbidity were correlated with median OS using a log-rank test. Correlation of surgery-related morbidity with the probability to receive adjuvant treatment was approximated by a Pearson Chi^2^ test. Tumor volume and EOR were correlated with OS with a Spearman-Rho test.

Additional analyses with regard to median EOR (Mann-Whitney-U test) and median MCS (Pearson-Chi^2^) between centers were performed. Surgery-related morbidity frequencies (Pearson-Chi^2^), PFS (log-rank test), and OS (log-rank test) between the two centers were noted in univariate analyses. Differences in patients’ OS between periods 1 and 2 were calculated utilizing a log-rank test, adjuvant treatment frequencies between inclusion periods were calculated with Pearson-Chi^2^.

**Surgeon experience**

The reference date for all survival calculations was the date of the primary tumor resection. First, the correlation analyses for the chosen outcome parameters (OS, EOR and surgery-related morbidity) were performed by using the actual number of surgeries performed before the index surgery. Secondly, the median values of the surgical experience data (i.e. number of prior surgeries) for all three time spans (i.e. lifetime, medium- and short-term experiences) were calculated and the experience data was then dichotomized by the median split for respective correlation analyses. Thus, surgeon experience was assessed as a continuous as well as categorical variable (i.e. median split).

Surgeon experience as a continuous variable was correlated with OS (Spearman’s rho test), EOR (Spearman’s rho test), and occurrence of surgery-related morbidity (Eta test). Analyses with the dichotomized data were correlated with OS (Kendall’s Tau test), EOR (Kendall’s Tau test) and surgery-related morbidity (Pearson Chi^2^).

A posthoc analysis dividing surgeon experience numbers into quartiles was performed. A comparison of the least experienced (<25 percentile) and the most experienced surgeons (>75 percentile) did not show a significant correlation with regard to the chosen outcome parameters, which is why quartile analysis was abandoned for the more robust median split groups. Survival data, tumor volume and EOR were assessed as continuous variables, the occurrence of surgery-related morbidity, MCS and tumor location were assessed as categorical variables, and patients’ age and preoperative KPS were assessed as both continuous as well as categorized variables. Multivariable testing for survival was performed using a Cox proportional hazard model (HR with 95% confidence interval). MCS was correlated with surgery-related morbidity via a Pearson Chi^2^ and the surgeons’ experience (Kendall’s Tau test).

All variables were tested for normality (Kolmogorov Smirnov test), and appropriate tests for further analyses were chosen. For non-normally distributed data, the interquartile range (IQR) was given instead of range.
