# Supplementary material for: Surgeon experience in glioblastoma surgery of the elderly—a multicenter, retrospective cohort study
Source: J Neurooncol. 2023 Jan 31;161(3):563–72. doi: 10.1007/s11060-023-04252-3 (PMC9992256; doi:10.1007/s11060-023-04252-3)
Supplement: Supplementary file 2 — Supplementary material 2 (DOCX 12.6 kb) [file 11060_2023_4252_MOESM2_ESM.docx]

**Supplement 2: Correlation analyses**

| **Dichotomized lifetime-surgeon experience data** | |
| --- | --- |
| **Factor:** | **P-value:** |
| Patient age | .772, Kendall’s Tau |
| Preoperative KPS | .557, Pearson Chi^2^ |
| Adjuvant treatment | .623, Pearson Chi^2^ |
| **Dichotomized medium-term surgeon experience data** | |
| **Factor:** | **P-value:** |
| Patient age | .206, Kendall’s Tau |
| Preoperative KPS | .295, Pearson Chi^2^ |
| Adjuvant treatment | .243, Pearson Chi^2^ |
| **Dichotomized short-term surgeon experience data** | |
| **Factor:** | **P-value:** |
| Patient age | .151, Kendall’s Tau |
| Preoperative KPS | .313, Pearson Chi^2^ |
| Adjuvant treatment | .862, Pearson Chi^2^ |
